# A Plan for Providing the Poor with Nurses in Times of Epidemic Sickness

**Published:** 1856-04

**Authors:** Edward H. Sieveking


### TRANSACTIONS

OF THE

### EPIDEMIOLOGICAL SOCIETY OF LONDON.

### Papers and Communications.

### A PROPOSITION TO SUPPLY THE LABOURING CLASSES WITH NURSES IN THE TIME OF EPIDEMIC AND OTHER SICKNESSES.

By EDWARD H. SIEVEKING, M.D., F.R.C.P.

[*Read before the Epidemiological Society*, April 3rd, 1854.*]

It is a point upon which probably all who have had much intercourse with the lower orders of society will agree, that illness is comparatively a much severer infliction upon them, than it is upon the members of those classes who do not live from hand to mouth; for this reason, that it immediately affects their means of obtaining a livelihood, and encroaches

* In consequence of the present paper, the Epidemiological Society appointed a committee to consider the plan proposed in it. Certain modifications have been made in the manner by which the scheme is proposed to be realised; but the committee have recently resolved that it is expedient to publish the original paper, in order more fully to show the arguments by which they have been influenced. The Earl of Shaftesbury has pledged himself to the committee to support their scheme; and as only an order from the Poor-law Board is requisite to carry it into effect, the committee earnestly hope that the labours of two years may soon be crowned with success. The committee, in addition to the testimony of medical men, can bring forward numerous masters of workhouses to prove that their scheme is not only theoretically good, but practically perfectly feasible. Without this explanation it would be difficult to understand the apparent discrepancies between the writer's statements and the propositions adopted by the committee, which were published in the Transactions of the Epidemiological Society for April 1855.

upon their daily income. The absence of providence among the working classes is a frequent subject of declamation; but in numerous instances, in which a proper forethought and due thrift had been exercised, the calculations are set at nought by the inroads of disease. The actual outlay occasioned by sickness is no less a serious difficulty than the consequent loss of employment; a loss that may be temporary, but very probably becomes permanent. The savings, if any, soon evaporate under the pressure of disease. When the savings are gone, and no new resources have been opened in the interval—and, unless by a *deus ex machinâ,* where are they to come from, when work is at a stand still?—the pawnbroker's shop yawns to receive its victims, and henceforward we see but a downward ruin. The hardest case is undoubtedly the one immediately alluded to, where the head of the family is prostrated by sickness. The argument applies more or less to the occurrence of illness in any member. If it is the mother, the domestic concerns are not attended to—the father comes home to discomfort, anxiety, and turmoil; if the child or children, the mother's strength is taxed beyond what she can bear, and in too many instances she loses health, though she may not be a direct loser of a daily income—and she, too, is cast upon the sick-bed. Medical men and clergymen can bear witness to the fact, that disease spreads among the labouring classes, in a degree entirely disproportionate to the influence of sanitary relations. No one is inclined to estimate these at a higher value than I am; but, assuming all hygienic demands to be satisfied, in regard to sewerage, watering, drainage, and ventilation, it is maintained, that there still remains an amount of removable, or rather preventible disease, for which other remedies must be sought than those offered by the most perfect sanitary laws.

These convictions were forced upon me at a time when, as physician to a metropolitan dispensary, I was in the daily habit of visiting the sick poor at their own homes; when I had constant opportunities of observing how closely the well-being of families depended upon the health of the individual members; and how frequently the gradual and progressive ruin of a family was connected with, and traceable to, the first appearance of disease at their doors. It is more particularly to the political economist that the fact should be a subject for earnest and serious reflection; for the loss of health in the working man is a loss to the community—indirectly, by deprivation of his labour; directly, by the cost entailed. This fact is most sensibly brought home to us by our poor and county rates. We are not, it is unfortunately true, rated for our hospitals; but the amount of poor-rates is enormously swelled, indirectly as well as directly, by the amount of disease preventible in the manner alluded to. The illness that commences by diminishing and arresting the daily supplies, terminates its career by throwing its victims on the parish for maintenance or for burial.

An instance will best explain these observations. Not long since, I was induced to visit the domicile of a patient who was under my care at St. Mary's Hospital, as an out-patient. He was a respectable, hard-working man, a coach painter; he was labouring under rheumatism of the extremities, and was unable to handle his brush, and therefore to earn the bread for the subsistence of his family. I found him surrounded by four young children, the youngest a baby; his wife had been previously taken ill, and was obliged to resort to a hospital. The man himself, left in sole charge of four young children, had to work by day, and nurse his tender offspring by night. Was it surprising that his overwrought constitution sunk under the accumulating anxiety and stretch of body and mind? He followed his poor wife to an early, and, as far as human eye could reach, unmerited grave; and the four children became the recipients of parish relief, the inmates of a workhouse. Here there was no hygienic measure at fault, in the medico-legal sense; and yet it was a case where, in the most vulgar and lowest sense, it was the interest of the community to have prevented the fatal issue. Is it necessary to calculate in £ *s. d.* the cost to the parish of this unhappy family—the burial of two parents, the prolonged maintenance of four children—in order to prove that proper assistance, rendered at the outset of disease, would in all probability have prevented much misery, and have saved a large sum of money to the community?

The instance to which I have alluded may be regarded as exceptional; but this is erroneous. I select it merely because it occurred recently, and because it happens to illustrate particularly well the point to which I am anxious to draw attention—the means of preventing or arresting the inroads of disease among the working classes, by supplying them with nurses. In providing such assistance, it is not intended to establish substitutes for the present medical attendants, in the guise of female doctors; but to secure the efficient execution of the directions given by the medical man, and thus materially to aid his labours. This would form another important element in preventive medicine; while the presence of a nursing attendant in the sick-chamber would obviate the necessity which now exists of a constant intercourse between the neighbours and the sick—the proof of much kindly feeling, but also a fertile source of disease by contagion. The nurse would therefore be a direct benefit, not only to the house or family affected, but also to the immediate neighbourhood.

The preceding remarks have not been directed towards exciting philanthropic commiseration with the sick poor, but are made mainly with a view to showing that it is the immediate interest of the higher classes, and of the governing bodies, to provide a remedy for evils which, directly or indirectly, affect themselves. It is confessedly low ground to take; but it is the only ground upon which it would be possible to secure that extensive and uniform co-operation, which, if the views expounded are correct, and the scheme to be proposed any way feasible, must be brought to bear. The advantages obtainable by an early arrest of disease among the lower orders have of late been palpably illustrated, by the beneficial results yielded by the house-to-house visitation in districts in which epidemic cholera had made its appearance. Here we have a casual and terrifying calamity arrested, by a measure as temporary as the inroads of the enemy; but we need only turn to the Registrar-General's reports, and the returns of the Health of Towns Commission, to convince ourselves that a vast proportion of all endemic and epidemic disease, and a fearful mortality, might be prevented, and their consequences obviated, by similar provisions, not of a temporary, but of a permanent character. I trust I may not be accused of making vague statements, if I appeal rather to the personal experience of individual medical men, clergymen, and others, than to statistics, in order to prove my position regarding the pecuniary saving necessarily effected by the prevention of disease. Men with larger means at their disposal, and with more knowledge of these matters than I can command, have failed in the attempt satisfactorily to reduce these items to numbers. Mr. Chadwick* observes, that of the pecuniary burdens created by the neglect of sanitary measures at large, including the cost of maintenance during the preventible sickness, any estimate approximating to exactness could only be obtained by very great labour, which does not appear to be necessary;

---

* Report on an Inquiry into the Sanitary Condition of the Labouring Population of Great Britain, 1842, p. 188.

and further,* that the public loss from the premature deaths of the heads of families is greater than can be represented by any enumeration of the pecuniary burdens consequent upon their sickness and death. If, therefore, almost insurmountable difficulties present themselves to calculating the sum total of pecuniary loss entailed upon the community by preventible disease, it follows that we have still less a satisfactory basis upon which to found a mathematical demonstration of the loss resulting from the one item of improper neglect at the first outset of disease, and during its continuance, among the labouring classes. It is only right to allude to this difficulty, to show that it has not been lost sight of.

I venture to assume as admitted, that we may, apart from what are commonly called sanitary measures, by providing nurses for the poor when incapacitated from following their ordinary avocations by illness, prevent a large amount of disease, and consequently effect a considerable pecuniary saving to the community. The next question which appears to suggest itself, relates to the character of the nurses to be supplied. It is important that no exaggerated estimate be formed, that we may not frustrate our intentions by making demands of a nature not to be met; we must not, therefore, think it necessary, nor would it be desirable, to supply to the labouring classes nurses of the same qualities, and with the same pretensions, as those we procure from the training institutions for the wealthy. No private charity could achieve an undertaking of the kind, if it were attempted; and it is very questionable whether, if it could be done, it would be most advantageous to the parties whom we desire to benefit. The nurse to be sent out to the sick poor must essentially be an assistant; she must be able and willing to superintend the little household, or to mind the children, or do anything that the limited circumstances of the parties may render necessary; she must be habituated to their ordinary scanty mode of living, and not be spoilt by intercourse with the servants and members of the higher classes for the performance of those menial duties which will devolve upon her, if she is properly to fill her post. She must be one of the class she visits; she must be able to appreciate their wants, and to regard herself, *pro tempore,* not as a luxury, but as an integral part and a necessity of the family into which she enters. That a well trained nurse, actuated by Christian benevolence

* Report on an Inquiry into the Sanitary Condition of the Labouring Population of Great Britain, 1842, p. 269.

and charity, may do all this, and more, is beyond all doubt; but we must employ such materials as are most likely to be forthcoming; and, though they may not be of the best quality, we shall be more likely, in the present condition of our social machinery, to obtain permanent results by availing ourselves of what exists, than by attempting an entirely new creation. It appears to follow that we must develope some scheme, of a practical character, adapted to existing institutions, by which it shall be possible, at a minimum primary outlay, to provide for the sick poor of the entire country nursing attendants, who may be easily accessible, whose knowledge of the duties to be performed, and position in life, will render them willing and acceptable agents; and further, that, if such a scheme can be proposed and carried out, we shall soon effect a large saving in money to the poorer as well as to the wealthier classes, while we shall remove an amount of misery and destitution affecting the former, which is amenable to no other treatment.

There are two main points of view in which this matter can be regarded—the politico-economical view, which has been urged; and the ecclesiastical view, upon which, more or less, the schemes of a similar nature are based, which we meet with in other countries.

If it were possible to found any proposition of the kind in Great Britain upon religious convictions only, or to establish a distinct framework analogous to that of the Sisters of Charity of Roman Catholic countries, it might perhaps eventually work better and more effectively: but we must discard such an attempt as utterly futile; the absence of anything like religious uniformity at once negatives the feasibility of such a plan; and it is only because the view we advocate appears in itself consonant with our national habits of thought and mode of action, that we also believe that it may be realised. At the same time, it is hoped that our scheme in no way excludes, but rather aids in, the development of those principles of Christian benevolence and charity, without which all our endeavours must fail to produce the fruit we wish to ripen; for, while avoiding anything likely to introduce differences of a sectarian character, there will be full scope for the drawing together more closely the different classes of society, and for consolidating and extending those principles of Christian brotherhood upon which modern civilisation is based. Several corollaries suggest themselves in connexion with this matter, which I pass over for the present; and I proceed to explain the practical solution of the problem which I have to offer.

Given, the existence of a large amount of sickness, loss of time, and consequent destitution among the labouring classes, throughout the country, unavoidable or irremovable under the present state of our institutions ;—

Given, a large increase of our national expenditure, of our poor and county rates, resulting from the prevalence and spread of disease among the labouring classes, in the shape of direct pecuniary relief, of burial fees, of the support of orphans, contributions to lunatic asylums, and the like ;—

Given, the necessity of providing for the removal of a certain, indefinable, proportion of these evils, by arresting incipient disease, or preventing its spread ; and lastly—

Given, the further necessity of doing so, by supplying throughout the country a staff of nurses competent to attend to the sick, under the direction of the medical men, as well as to aid the respective families in their domestic avocations :—

It is sought to show that this may be done, by an adaptation of existing machinery, at a comparatively trifling primary cost, and with the certainty of large benefits of a pecuniary, sanitary, and moral nature.

The projected remedy is this : To employ the able-bodied men and women, that are now maintained in the workhouses, under certain necessary rules and restrictions, for the purpose of nursing ; and to make the workhouses both the nuclei of the system, and the places to which the parties requiring nurses may apply.

By the returns of the Poor-Law Commissioners, we learn that there are a certain number of able-bodied men and women in every workhouse in England and Wales. From the Fourth Annual Report of the Poor-Law Board, for 1851, I find that on an average, as shown in the subjoined table,

| No. of Unions in England. | Total of Persons relieved by Unions. | Out-door Paupers. | | | Able-bodied in Unions. | |
|---|---|---|---|---|---|---|
| | | Total. | Not able-bd. | Lunatics. | Males. | Females. |
| 553 | 761,216 | 658,594 | 329,810 | 8,319 | 8,269 | 12,868 |

Average number of able-bodied men in each union, about .... 15
Average number of able-bodied females in each union, about . 23

there are fifteen men and twenty-three women of this class in every union in England. They are now useless members of the community ; they, like the other inmates of our workhouses, are even less the objects of Christian philanthropy than the denizens of our prisons. Labouring under the stigma of pauperism, they seem cast out of the pale of society. By giving them a suitable and useful occupation, we raise their moral dignity, while we confer a benefit upon the community at large. The practical difficulties—independent of any obstacles that may present themselves on the part of the poor-law guardians—are, that these people perhaps require more training than the workhouse infirmaries may afford, and that some prejudices must be overcome on the part of the classes to whom they are to be sent. But surely these are not insurmountable impediments; the former may be remedied by the appointment of a superintending nurse or matron, by training in the infirmaries, under the supervision of the guardians of the poor, the clergy and medical men of the union; the latter must be overcome by the spread and force of public opinion, which in this instance would mainly depend upon the countenance afforded by the medical practitioners of the neighbourhood. Our knowledge of human nature, and the special experience offered by provident institutions of all kinds, teach us that we should not offer our boon gratuitously, except where it is unavoidable; the saving to the individual families, it is apprehended, would be sufficient to enable them to remunerate the nurse, or to pay a sum to the union for her services. A minimum and maximum rate might be fixed in different localities; and by this means we might be enabled to offer to the deserving nurses a bonus to stimulate them to good behaviour. Thus an incentive would be held out, which would prevent the nurse from regarding her work as forced drudgery; and she would discharge it with a very different *animus*, than if no benefit could accrue to her from it, beyond the scant praise of her workhouse superiors. Until the system had been brought into working order, the aid of the clergy and of visiting societies would be specially invoked, to establish a more minute and careful supervision of the workhouse nurses than the paid officials alone could carry out.

It is here that I would briefly allude to the corollaries which seem deducible from the system proposed, and to which I have above adverted. It is hoped that, by introducing a feeling of competition, and a prospect of independence, among the pauper inmates of unions, their *morale* will be raised; and that the hopeless condition, which establishes a close analogy between these monuments of modern political economy and Dante's *Inferno*, may be ameliorated. Thus a benefit will be conferred upon the individual through whom it is desired to benefit others, as well as upon the community. It is probable that the emulation will extend beyond the workhouse doors, and that, by supplying partially the want of nurses, many respectable females may be induced to devote themselves to one of the most honourable pursuits for which females are fitted; and that, in the course of time, a discreditable nurse may be what she now unfortunately is not, a *rara avis in terris*. Should in this way eventually the employment of workhouse nurses be rendered unnecessary, it could only be a matter of rejoicing; the object would be gained more fully than can now be anticipated. It is not unreasonable to assume that indirect beneficial results may arise from the system, if well supported and efficiently carried out. "Our workhouses," as I remarked in a pamphlet published on the same subject in 1849, "our workhouses have not hitherto met with that attention on the part of the higher ranks, and more especially of the visiting ladies, which they deserve. An intimate acquaintance with the necessities, moral and physical, of the inmates, will lead to a better knowledge of the relations and wants of the poor generally, and encourage kindly feeling on all sides. Everything done to raise the *morale* of the poor, will react beneficially upon the wealthy classes; and will tend to diminish the amount of crime on the one hand, and the poor and county-rates on the other."

It was originally proposed to apply the scheme only to the able-bodied females residing in workhouses. A gentleman, formerly in large practice in a country district, who in the main coincided with the views which have been submitted to your notice, has informed me that it would be a great advantage to country practitioners if, in certain cases, they could obtain male assistance; he alluded to delirium and insanity, and stated that two or more able-bodied male nurses in an union, for out-door purposes, would find ample employment in this way. There can be no doubt that many similar suggestions might be offered, and would prove very salutary, if the subject were fairly grappled with. I now merely wish to contend for the principle; the mode of realising it must necessarily vary in different localities. The labour requisite to carry out so extensive a plan is greater than an humble individual like myself, with the cares of a work-day life, can cope with. I can do little more than solicit the kind attention of my professional brethren to the subject. For five years it has been uppermost in my heart; and the earnest conviction that society is called upon to provide a remedy of the kind suggested—a conviction only strengthened by time and reflection and inquiry—has given me courage to submit the subject to this learned assembly. As a subject closely allied to one of the professed purposes of the Epidemiological Society, the prevention of disease, it appears to have a special claim upon the consideration of the members. I only entreat that they will not cast the proposition aside on account of the desultory manner and feeble voice of the advocate, but that they will decide upon the question at issue by the light of their own experience. If their verdict be in favour of the scheme, there is much hope that it will prosper—that the seed will germinate and spring into vigorous life. Most earnestly do I hope that such may be its fate.

